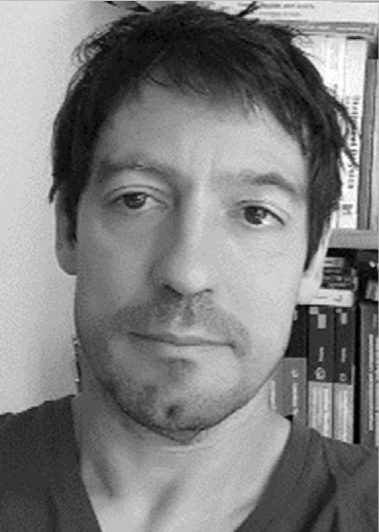# Atomic Force Microscopy Meets Biophysics, Bioengineering, Chemistry, and Materials Science

**DOI:** 10.1002/cssc.201802383

**Published:** 2019-01-22

**Authors:** José L. Toca‐Herrera

**Affiliations:** ^1^ Institute for Biophysics, Department of Nanobiotechnology University of Natural Resources and Life Sciences Vienna (BOKU) Muthgasse 11 1190 Vienna Austria

**Keywords:** atomic force microscopy, materials science, natural products, scanning probe microscopy, surface analysis

## Abstract

Briefly, herein the use of atomic force microscopy (AFM) in the characterization of molecules and (bioengineered) materials related to chemistry, materials science, chemical engineering, and environmental science and biotechnology is reviewed. First, the basic operations of standard AFM, Kelvin probe force microscopy, electrochemical AFM, and tip‐enhanced Raman microscopy are described. Second, several applications of these techniques to the characterization of single molecules, polymers, biological membranes, films, cells, hydrogels, catalytic processes, and semiconductors are provided and discussed.

## 1 Introduction

According to Web of Science, 104 522 items are associated with the term “atomic force microscopy” (AFM) since 1987 (search carried out in December 2018). This large number shows that AFM is well established in the scientific community. After refining the search, the number of items related to both scientific and technological fields, for example, materials science multidisciplinary (32 389), applied physics (28 921), chemistry physical (21 175), physics condensed matter (14 762), and chemistry multidisciplinary (13 887), is still large.

AFM,^1^ as part of the scanning probe microscopy family of techniques, has been utilized for the last three decades to characterize (bio)molecules and (bio)materials.^2^, ^3^, ^4^, ^5^, ^6^ In comparison with other characterization techniques, such as electron microscopy (transmission and scanning), AFM seems to be more versatile. For example, it permits dynamic processes to be followed at the micro‐ and nanoscale in aqueous environments at different temperatures. In addition, AFM can be combined with other characterization techniques, such as fluorescence microscopy or Raman/IR spectroscopy, to overcome its own limitations (e.g., absolute distance or chemical fingerprint determination).^7^, ^8^

For further reading, the following reviews summarize the versatility and measuring properties of AFM in different fields. Francis et al. discussed the contribution and versatility of the technique applied to biological and biomedical systems.^9^ Handschuh‐Wang et al. described, in a compact way, new advances in the combination of optical techniques with AFM, explaining their basic principles and pointing out different applications.^10^ An extensive article presenting the history, development, and prospects of high‐speed AFM was written by Ando.^11^ Researchers interested in high‐vacuum AFM should focus on the detailed article written by Giessibl.^12^ Patel and Kranz,^13^ in a very complete work, discussed how tip modification delivered chemical information. They also explained the use of electrochemical imaging and their applications. Finally, Gross et al. recently reported progress and challenges of high‐resolution scanning probe microscopy with functionalized tips in the elucidation of molecular structures.^14^

## 2 AFM‐Based Characterization Techniques

### 2.1 Topography imaging and mechanical machines

**Figure 1:**
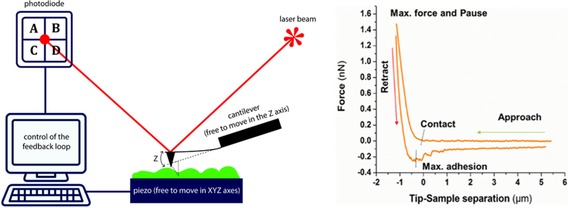
Left: Main component of an AFM setup. A flexible cantilever (in this case, with a sharp tip) interacts with the sample (through attractive/repulsive forces), and therefore, is the AFM sensing element. The deflection of the cantilever due to interactions is measured after detecting the reflected laser beam with a position detector (a four quadrant photodiode). The piezoelectric scanner can be moved with nanoscale resolution along either the *x*–*y* or *x*–*y*–*z* axes, depending on the commercial device. Right: Representative force–distance curve recorded for a living cell. The measurement starts with the cantilever at rest (sensing zero force). Then, the cantilever approaches the cell to deliver information about molecular and colloidal forces. Finally, the cantilever is retracted from the sample to convey information about the existence of adhesive forces, molecular unfolding events, and tethers. (Image adapted from Ref. 33.)

If spectroscopy and optical techniques are based on the interaction between light and matter, AFM is mainly defined by the interaction of matter with matter. Briefly, the main elements of an AFM are a scanner, probe (a bare cantilever or a cantilever with a sharp tip or a colloidal particle), photodetector, and computer (Figure 1). The interaction forces acting between the tip and sample bend the cantilever according to the Hooke law. Bending is monitored and detected by a photodiode that collects the reflection of a laser beam at the backside of the cantilever, while scanning or recording force curves. A piezo scanner moves either the sample or the tip in three dimensions. The surface properties of the sample are then obtained from the tip/sample interaction and the final image is delivered by a computer.

Generally, the most used imaging modes are contact and tapping mode. Other researchers refer to static and dynamic modes with all of their possibilities: contact, jumping, tapping, amplitude, and frequency modulation. An important advance in scanning is high‐speed AFM, which was developed by Ando et al.,^15^ and deserves a review on its own.

In contact mode, while the sample is scanned, the value of the repulsive force between tip and sample is kept constant. A gentler scanning option, especially for soft samples, is tapping mode because it reduces lateral forces that could damage the samples. In this mode, the cantilever is set to oscillate vertically at (or close to) its resonant frequency. Once the tip is far away from the sample, the cantilever oscillates with constant amplitude, whereas for smaller tip–sample distances the amplitude of the oscillations is reduced. High‐resolution images can be obtained by using a feedback loop that keeps the amplitude of the cantilever oscillation at a constant level during scanning. Differences between the set drive phase and phase of the cantilever response can be used to gain an insight into viscoelastic and adhesive properties of the sample under study. Interesting and didactic literature concerning different AFM measuring modes can be found in the work of several authors.^16^, ^17^ Representative (biological) samples measured with these imaging modes include nanotubes, lipids, proteins, molecular self‐assemblies, or cells.^18^, ^19^, ^20^, ^21^, ^22^

At this point it is worth mentioning other methods for beam‐deflection measurements implemented by research groups with a long tradition and expertise in AFM development. Among them, the most relevant ones are capacitive detection by using cantilevers as capacitors (their deflection produces a change in capacitance)^23^ and piezoelectric detection (for which the deflection of piezoelectric cantilevers is detected as an electrical signal; this permits atomic resolution to be achieved).^24^

Apart from scanning, AFM opens up a wide range of experimental possibilities upon use as a “mechanical” machine; this is technically known as force spectroscopy. In this case, force–distance (or force–time) experiments are carried out. In such experiments, the AFM tip (or a colloidal probe) approaches and is retracted from the sample at different speeds (which might range from 30 to 10 000 nm s^−1^). Bending of the cantilever is determined as a function of the displacement of the piezo scanner, whereas the force sensed by the cantilever is calculated based on Hooke's law (the bending or deflection of the cantilever is multiplied by the spring constant).^25^ The force–distance curves can be divided into three parts (see Figure 1, right). First, the approaching curve delivers information about repulsive or attractive forces (e.g., electrostatic, van der Waals, hydration, or entropic forces).^26^, ^27^, ^28^ Second, contact of the cantilever with the sample (the dwell time can be defined by the researcher) permits mechanical properties (e.g., the Young modulus, relaxation time, and viscosity) to be investigated.^29^, ^30^, ^31^ Finally, the retracting curve provides information about nonspecific adhesion forces, ligand–receptor forces, tethers, and molecular unfolding.^3^, ^32^, ^34^ To quantify the interaction forces, the spring constant of the cantilever has to be evaluated before starting the experiments, whereas the contact point between tip and sample has to be determined for every recorded force curve.^35^, ^36^

Modification of the tip (or colloidal particle) through well‐defined chemistry leads to a particular type of force spectroscopy (chemical force microscopy) and a new form of imaging based on molecular recognition.^37^, ^38^ In both cases, “two molecules” interact specifically. These experiments permit the study and determination of particular interactions (e.g., hydrophobic–hydrophobic, ligand–receptor).

A breakthrough in high‐resolution imaging is the work of Schuler and co‐workers.^39^ They were able to resolve the structure at an atomic level of asphaltene molecules by combining AFM with scanning tunneling microscopy. Such results opened up the possibility of investigating, in detail, the structure of compounds used in molecular electronics or photovoltaic devices.

### 2.2 Electrical modes (Kelvin‐probe force microscopy and electrochemistry)

**Figure 2:**
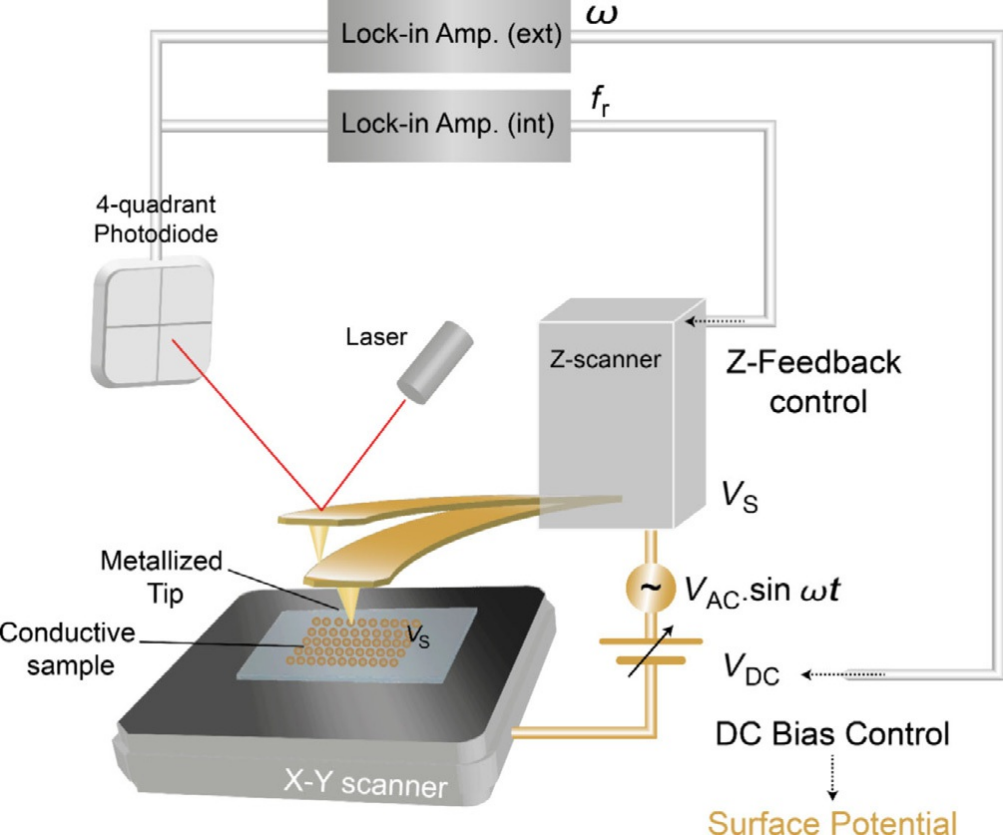
In KPFM, an alternating current (AC) plus direct current (DC) voltage is applied to the cantilever, causing an oscillating electrostatic force between tip and sample. The resulting deflection oscillation is detected with a lock‐in amplifier and minimized by the DC voltage, which equals the local contact potential difference between tip and sample (*f*_r_: resonant frequency; *ω*: applied frequency; *V*_s_: surface potential; *t*: time). (Figure adapted from Ref. 85.)

Another interesting possibility is to use conducting probes (tips or particles) to combine classical scanning with electrical surface mapping. This is the case for Kelvin‐probe force microscopy (KPFM). With this technique, the conducting cantilever is scanned over a sample at constant height to obtain the contact potential difference, *V*_cpd_ (which is determined by the work functions of both tip and sample; Figure 2). Measurement of the contact potential difference is performed by applying an oscillation (of frequency *w*) in the bias voltage to the probe and sample. In this way, a capacitor is formed. Simultaneously, the cantilever is oscillated at its resonance frequency, *ω*_0_, and a typical noncontact contact mode feedback acts on the phase, so that the frequency shift can be monitored and registered. If the dependence of the bias voltage with time is *V*_bias_=*V*_DC_+*V*_AC_ sin (*wt*), with steady DC and oscillating AC components, the frequency shift exhibits two harmonic components (at *ω* and 2 *ω*) of expressions: Δ*F*_w_≈(*V*_DC_−*V*_cpd_)*V*_AC_ sin (*wt*) and Δ*F*_2w_≈(*V*_AC_)^2^ cos (*wt*). Now, if a second feedback mechanism is able to change *V*_DC_ such that Δ*F*_w_=0, *V*_DC_ will be equal to the contact potential difference, *V*_cpd_, which is the magnitude of interest. In general, mapping of the work function provides information about corrosion phenomena, catalytic activity, or electrical properties of junction devices.^40^, ^41^, ^42^

The last measuring mode discussed in this section is related to electrochemical experiments. By adding a special sample holder with three electrodes to the standard AFM configuration, it is possible to follow redox reactions that are taking place in electrolyte solutions on an electrode surface, as well as to characterize the morphology of the surface of the electrode.^43^ Basically, the sample acts as a working electrode, while the nonconducting tip monitors variations in the sample as a function of time. Experimentally, it is recommended to use dilute electrolyte solutions and to avoid corrosion effects on the AFM scanner and AFM tip. A contemporary extension of this measuring mode is the use of a special metal‐coated tip that is insulated, except at its apex, which is utilized as an electrode to detect species in an electrolyte solution.

### 2.3 Spectroscopy mode (tip‐enhanced Raman spectroscopy)

**Figure 3:**
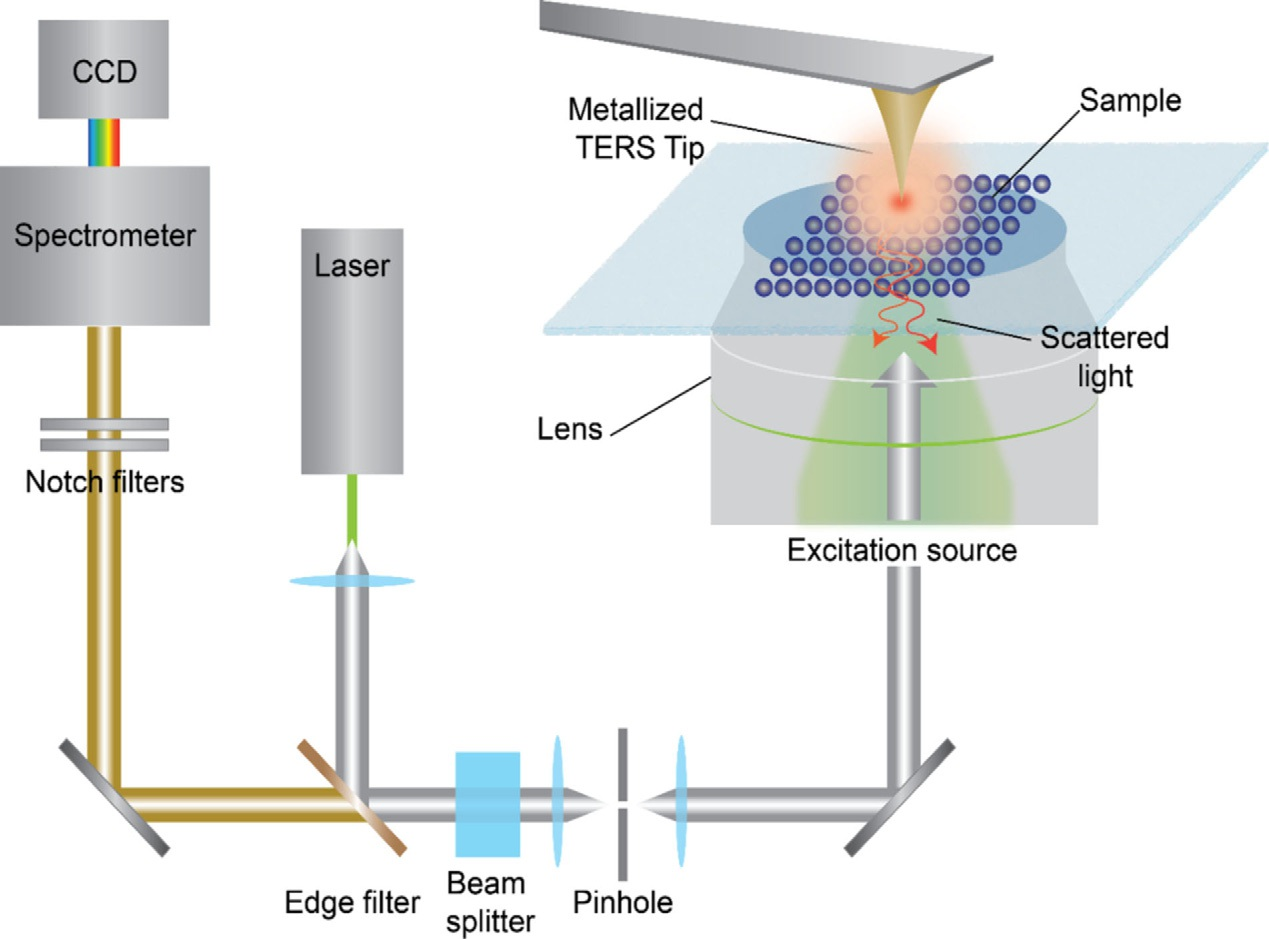
In TERS, a sharp tip (made of Au or Ag) works as an antenna. Once the laser is aligned at the correct location, the sample stage scans the sample (in this way, laser alignment onto the tip does not change). Raman spectra emitted by the sample are collected by a spectrometer (and a charge‐coupled device (CCD) camera).

Raman spectroscopy permits vibrational and rotational modes to be monitored, providing a structural fingerprint by which molecules can be identified.^44^ Therefore, the combination of AFM (which can be operated in liquid conditions) with Raman spectroscopy can be useful to investigate both topographical and chemical changes at the nanoscale for a wide range of (biological) samples, such as fibril proteins,^45^ carbon nanotubes,^46^ DNA,^47^ and cells.^48^ More specifically, tip‐enhanced Raman spectroscopy (TERS)^49^ uses an apertureless probe to enhance the Raman signal emitted by the sample molecules, which are separated from the probe by a few nanometers, and a metallic tip that is irradiated along its apical axis by a laser with a wavelength in the visible region (*λ*=500–650 nm). Because the tip is sufficiently close to the sample, field enhancement is possible, leading to molecular excitation and registration of local Raman spectra. Field enhancement is related to excitation of metal plasmons of the tip, which acts as an antenna. The maximum excitation range is about 20 nm in the vertical direction and 20–50 nm in the lateral one. Once the laser is aligned to the tip (its wavelength should match the resonance of the surface plasmons), the sample stage scans the sample underneath the tip, without disturbing the initial laser alignment on the tip. TERS can be used in different setups, and therefore, is able to operate in either reflection (convenient for nontransparent samples) or transmission (this mode needs an inverted microscope) modes. Figure 3 shows a typical transmission TERS configuration.

## 3 Selected Examples of Applications

Although the previous methodological section contains basic references to introduce the reader to different measurement possibilities of AFM, in this section, a collection of articles (many of them published in the last four years), considered to be scientific and technically interesting for the reader of *ChemSusChem*, have been selected. The section is divided by (model) system and contains different examples that were investigated with the four methodologies described in the previous section (high‐resolution imaging, force spectroscopy, electrical modes, and tip‐enhanced Raman spectroscopy).

### 3.1 Molecules and polymers

**Figure 4:**
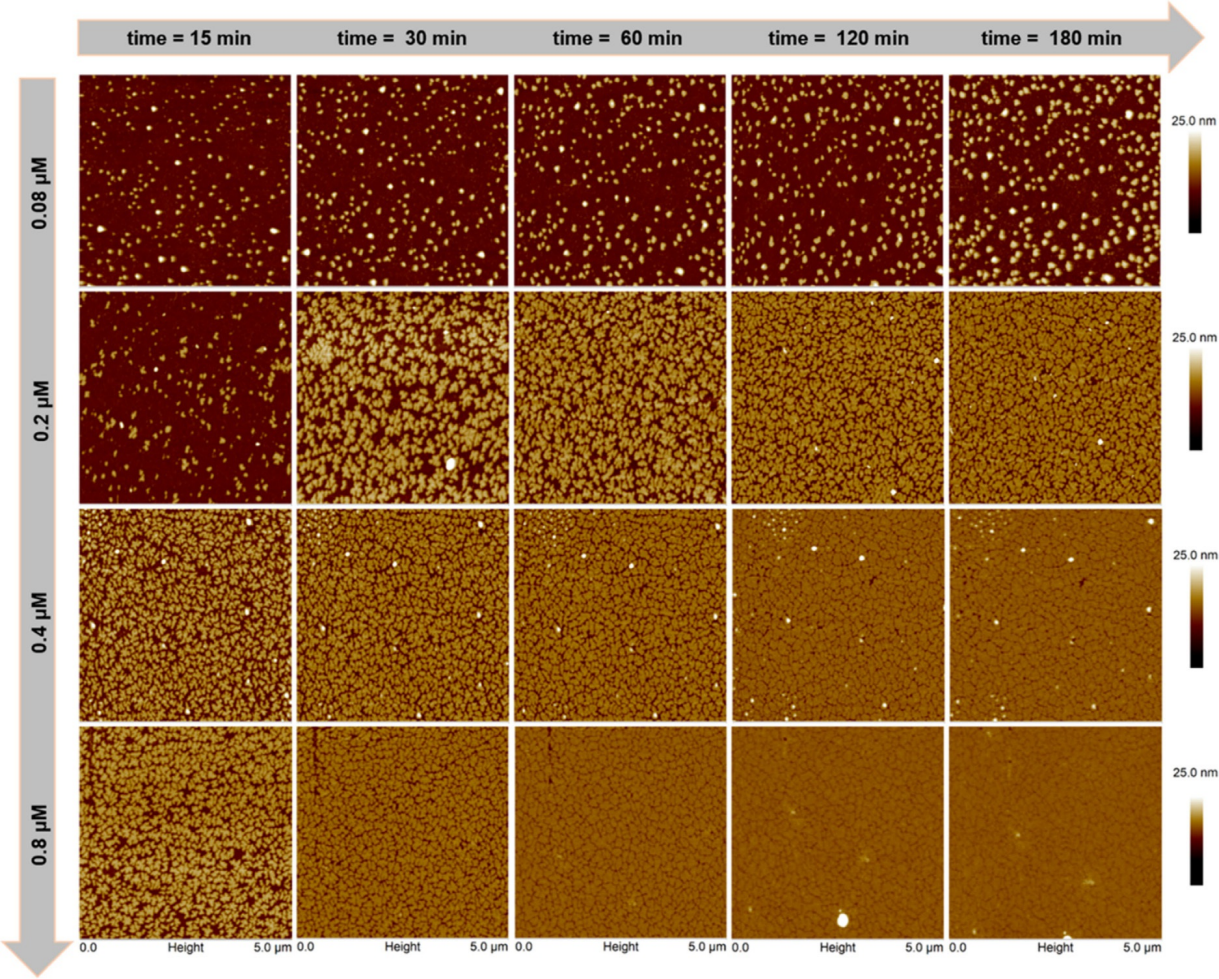
Two‐dimensional protein crystal growth as a function of time (*x* axis) and protein bulk concentration (*y* axis). Standard AFM (tapping mode) imaging is able to register coverage of the fluoride‐functionalized hydrophobic SiO_2_ surfaces. Experimental adsorption data can be used to check growth models and to optimize parameters for protein layer formation. (Image taken from Ref. 52, with permission.).

A recent example of the strength of AFM, in terms of high resolution, has been reported by Buchholz and co‐workers.^50^ The authors, by combining AFM with circular dichroism (CD) spectroscopy and dynamic light scattering, investigated the blood protein beta 2‐glycoprotein, which presents two main conformations (open and closed). AFM images revealed that lysine acetylation promoted a larger population of proteins in an open conformation, which played a role in the autoimmune disease antiphospholipid syndrome. Another example of the resolution capability of the atomic force microscope can be found in the work of Milhiet et al.^51^ In this study, a new method to locate transmembrane proteins in lipid bilayers was tested with AFM. The authors could demonstrate that proteins were incorporated within the lipid bilayer through their hydrophobic domains. Other dynamic processes can be also monitored with standard AFM imaging. Moreno‐Cencerrado et al. followed the formation of 2D bacterial proteins crystals as a function of time for different protein concentrations (Figure 4).^52^ Such measurements enabled the testing of theoretical crystal‐growth models.

A very instructive work concerning protein immobilization strategies for performing single‐molecule force spectroscopy experiments has been presented by Becke and co‐workers.^53^ They provided a protocol that described the covalent binding of proteins to silicon surfaces by straightforward chemistry (e.g., the use of silanes, 1‐ethyl‐3‐(3‐dimethylaminopropyl)carbodiimide and *N*‐hydroxysuccinimide). Furthermore, they determined the interaction force (about 50 pN) between adhesin RrgA (from *Streptococcus pneumoniae*) and fibronectin. This study shows the relevance of molecular orientation in force spectroscopy measurements.

The combination of force spectroscopy measurements with tips that are chemically modified permits recognition experiments to be performed on model systems. The work of Parreira and co‐workers illustrated the power of this approach for studying bacterial adhesion.^54^ They reported on the binding force between adhesin BabA (from *Helicobacter pylori*) and a glycan receptor. The strategy used involved the immobilization of BabA on the AFM tip, while the glycan receptor was linked to biotin self‐assembled monolayers. This study is also interesting because of analysis of the theoretical bond kinetics. Hence, the main findings showed that two bond populations described the binding process.

Among recent work on the mechanical unfolding of proteins, the study performed by Yu et al. is remarkable.^55^ They reported on instrumental limitations that might not allow existing intermediate states to be detected while performing force spectroscopy measurements. Through optimization of the experimental setup, it was possible to obtain microsecond resolution. In this way, the authors could mechanically unfold bacteriorhodopsin molecules in native lipid bilayers to find new intermediate states. Their setup permitted the unfolding of about two amino acids to be observed over microseconds (usual experiments record the unfolding of 6 to 60 amino acids over milliseconds). Furthermore, they were able to reconstruct the folding free‐energy landscape.

Recently, TERS was utilized to characterize protein glycosylation and glycans.^56^ The authors pointed out the complexity of data processing (e.g., multivariate analysis) because of the potential dependence on the orientation of the protein. Furthermore, the results indicated that the technique could discriminate between native and glycosylated forms of RNase.

TERS has also been successful in the detection of nucleobases. Treffer and co‐workers performed experiments on single‐stranded adenine and uracil polymers.^57^ Their results constitute an advance because spectral contributions of the nucleobases could be distinguished on a strand. In addition, if the tip was moved laterally in steps of a base‐to‐base distance, the collected spectra gave sequential information.

### 3.2 Membranes, fibers, and cells

Supported lipid bilayers are one of the most studied model membranes. For this particular case, AFM imaging has offered many possibilities to researchers. One of the latest published works deals with the monitoring of phase transitions of lipid mixtures of 1‐palmitoyl‐2‐oleoyl‐*sn*‐glycero‐3‐phospho‐(1′‐*rac*‐glycerol) and 1‐palmitoyl‐2‐oleoyl‐*sn*‐glycero‐3‐phosphoethanolamine (POPG:POPE). Borrell et al. reported on differences in the shift of the melting transition for liposomes (measured in bulk through differential scanning calorimetry) and lipid bilayers (observed through AFM).^58^ Recently, Unsay et al. published an interesting academic work on the use of AFM imaging and force–distance curves on lipid bilayers.^59^ The authors described a protocol to build supported lipid bilayers on solid supports (e.g., mica), and reported on the topography and mechanical properties of (1,2‐dioleoyl‐*sn*‐glycero‐3‐phosphocholine (DOPC)/sphingomyelin (SM)/cholesterol (Chol)) lipid bilayers. AFM, in combination with total internal reflection fluorescence microscopy, was used to image, in real‐time, cellulose hydrolysis on nanometer‐scale fibers.^60^

In this regard, AFM has delivered important information about cellulose fibers. Lahiji and co‐workers investigated the topography and elastic and adhesive properties of wood‐derived cellulose nanocrystals under different humidity conditions.^61^ They implemented finite element calculations to estimate a transverse elastic modulus in the order of GPa, and found variations in thickness from 3 to 8 nm. Another interesting and more focused study on the elasticity of cellulose was performed by Hellwig et al,^62^ who determined the mechanical properties of wet cellulose beads of different charge densities by recording force–distance curves with a colloidal probe (i.e., gold particles of about 20 μm). The resulting data were computed by means of the Derjaguin–Müller–Toporov (DMT) model, which included the adhesion force between the sample surface and AFM probe in the analysis.

Investigating a more complex system, Muraille et al. indented one type of lignified cell wall and compared the results with those obtained from lignocellulosic films; thus showing the role of lignin on the mechanical properties of cell walls.^63^ Another work concerning the mechanical characterization of wood cell walls was carried out by Cassdorf et al.,^64^ who could distinguish between the cell wall layers of the compound middle lamella of spruce wood by means of mechanical measurements.

For about 20 years, AFM (imaging and force spectroscopy) has also been applied to bacteria and human cells. Recently, El‐Kirat‐Chatel et al. investigated the adhesion of bacteria (which presented different phenotypical traits) on antifouling coatings to be used on ship hulls.^65^ The main findings indicated that adhesion forces at the population level did not always correlate with individual adhesion forces because some bacteria were susceptible to phenotypic heterogeneity amid their population. If one considers bacteria as colloidal objects, the surface potential could play an important role, through electrostatic interactions, on adhesion phenomena. Such surface potential (or surface charge density) can be determined by means of KPFM. Birkenhauer and Neethirajan illustrated how to characterize the surface potential of *Staphylococcus aureus* on steel and gold surfaces (functionalized with poly‐l‐lysine).^66^

**Figure 5:**
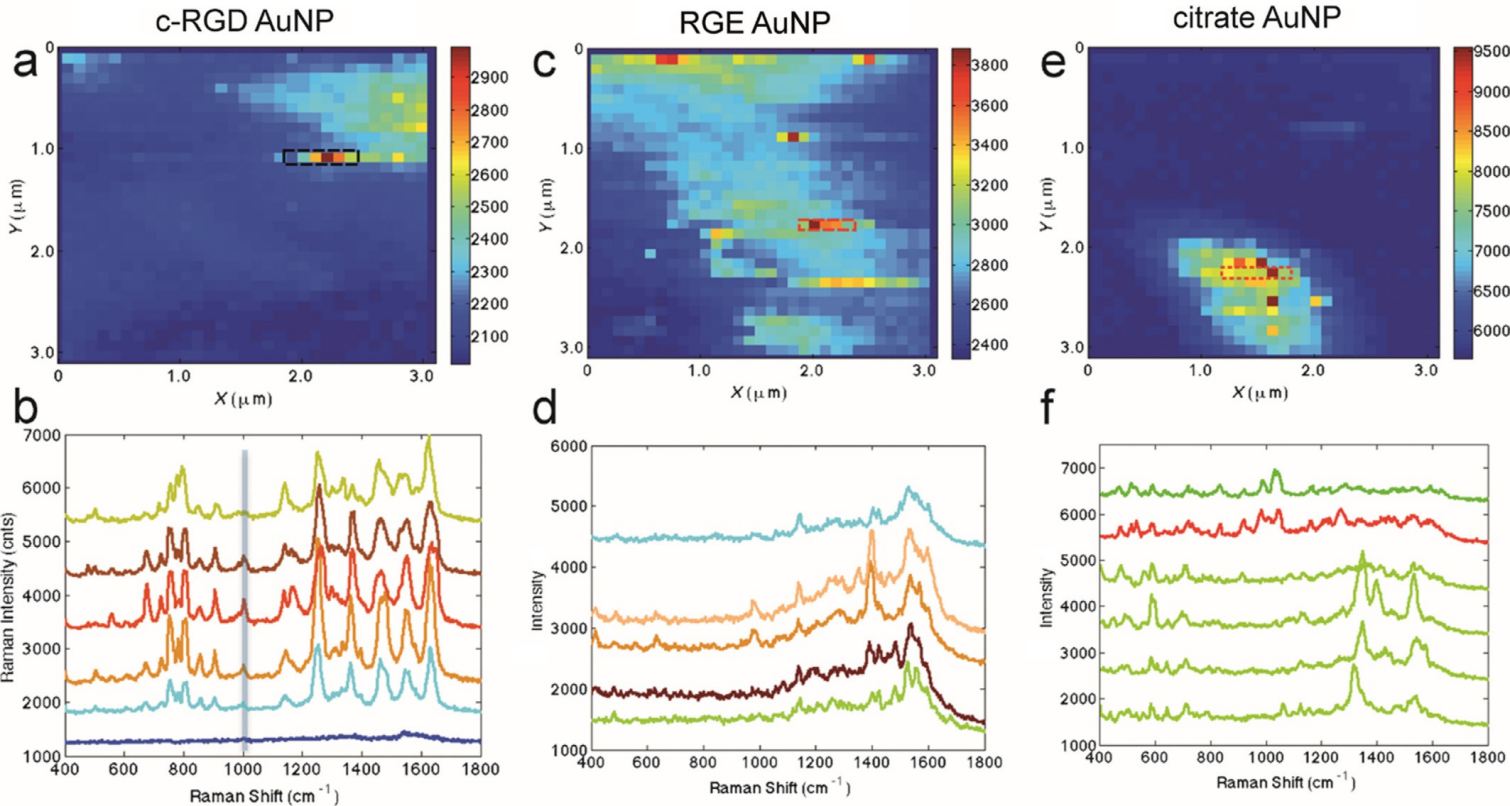
TERS intensity maps (top) and corresponding TERS spectra (bottom) of cells incubated with three functionalized NPs: c‐RGD–AuNP (a, b), RGE–AuNP (c, d), and citrate–AuNP (e, f); RGE=arginine–glycine–glutamic acid–phenylalanine–cysteine, AuNP=gold nanoparticles. The heat map was obtained from the vertical line near 1003 cm^−1^. Although the spectra of RGE and citrate NPs showed wide variation, the spectrum of c‐RGD–AuNPs was reproducible, possibly due to the integrin receptor. (Figure taken from Ref. 71, with permission.)

Imaging and force spectroscopy also provided interesting results in the investigation of human cell lines. Nowadays, it can be said that AFM is a diagnostic tool, which can differentiate healthy from unhealthy cells.^67^ A recent and very complete work on cell mechanics was performed by Efremov and co‐workers.^68^ The authors proposed a method to acquire the viscoelastic properties of cells (and hydrogels) for arbitrary loading. They tested the method on different cancerous cell lines and induced transitions from epithelial to mesenchymal states, and performed finite element simulations to model and validate the experimental results. Force spectroscopy at cellular level can be used to elucidate the main molecules responsible for the adhesion of bladder cancer cells to endothelial cells. Duperray et al. quantified adhesion forces and identified the key ligands involved in the process.^69^ Complementary experiments to target membrane receptors in healthy and cancers cells can be carried out with TERS. In particular, Xiao and Schultz showed that TERS could differentiate between different membrane receptors (integrins) that bound to the same arginine–glycine–aspartate–phenylalanine–cysteine (RGD) sequences.^70^ In a previous study, Wang and Schultz demonstrated that the Raman spectrum of the α_v_β_3_ integrin receptor could be detected in the membrane of colon cancer cells.^71^ The measurement strategy consisted of exposing the cells to nanoparticles (NPs) functionalized with cyclic RGD (c‐RGD) ligands before collecting the Raman signal. Figure 5 shows the variation of the TERS intensity and spectra for particle/cell interactions.

### 3.3 Hydrogels, catalysis, corrosion, and semiconductors

Interest in hydrogels has increased in recent decades due to their “smart” response to changes in environment (e.g., temperature, ionic strength, humidity). Researchers working on tissue engineering,^72^ plasmonics,^73^ or biosensing and drug delivery^74^ have taken advantage of the physicochemical properties of hydrogels. Vamsi and Yadavalli characterized the mechanical properties of poly(ethylene glycol) diacrylate based hydrogels, which are important for biomedical applications, at the micro‐ and nanoscale.^75^ The authors quantified the effect of monomer molecular weight, initiator concentration, and hydration rates on the mechanical properties of the hydrogels through force–distance curves.

Catalysis is important for scientific and technological fields such as biology, medicine, environmental chemistry, or chemical engineering. The study of catalytic phenomena at the micro‐ and nanoscale requires a combination of AFM scanning and spectroscopy. Harvey and co‐workers integrated Raman spectroscopy with AFM to study the photo‐oxidation of rhodamine 6G over Al_2_O_3_‐supported AgNP.^76^ The experiments were carried in a cell in which gas and temperature could be varied. A recent article on electrochemical AFM features demonstrated how a conducting tip sensed the electronic properties of cobalt (oxy)hydroxide phosphate.^77^ Another use of electrochemical AFM was its application in an investigation of corrosion on aluminum alloys in solution. Davoodi et al. proposed a probe that could work either as cantilever or as microelectrode. In their experiments, the electrochemical current was registered by using the redox mediator I^−^/I^−^_3_.^78^

Recently, studies that extend an understanding of heterogeneity on graphite/graphene surfaces for electrochemical applications have been conducted by Nellist et al.^79^ They used a nanoelectrode (with a conical Pt tip) in combination with tapping AFM mode to characterize surface topography, nanomechanics, and nanoelectronic properties.

KPFM has also been a useful tool to investigate photovoltaics and solar conversion technologies. In both cases, high energy conversion efficiency and low‐cost processing are key goals. In this context, Jiang et al. investigated charge transport and separation in perovskite solar cells.^80^ Their results indicated the existence of a p–n junction structure at the TiO_2_/perovskite interface. The results also showed that improved carrier mobility was essential to increase the efficiency of perovskite solar cells.

**Figure 6:**
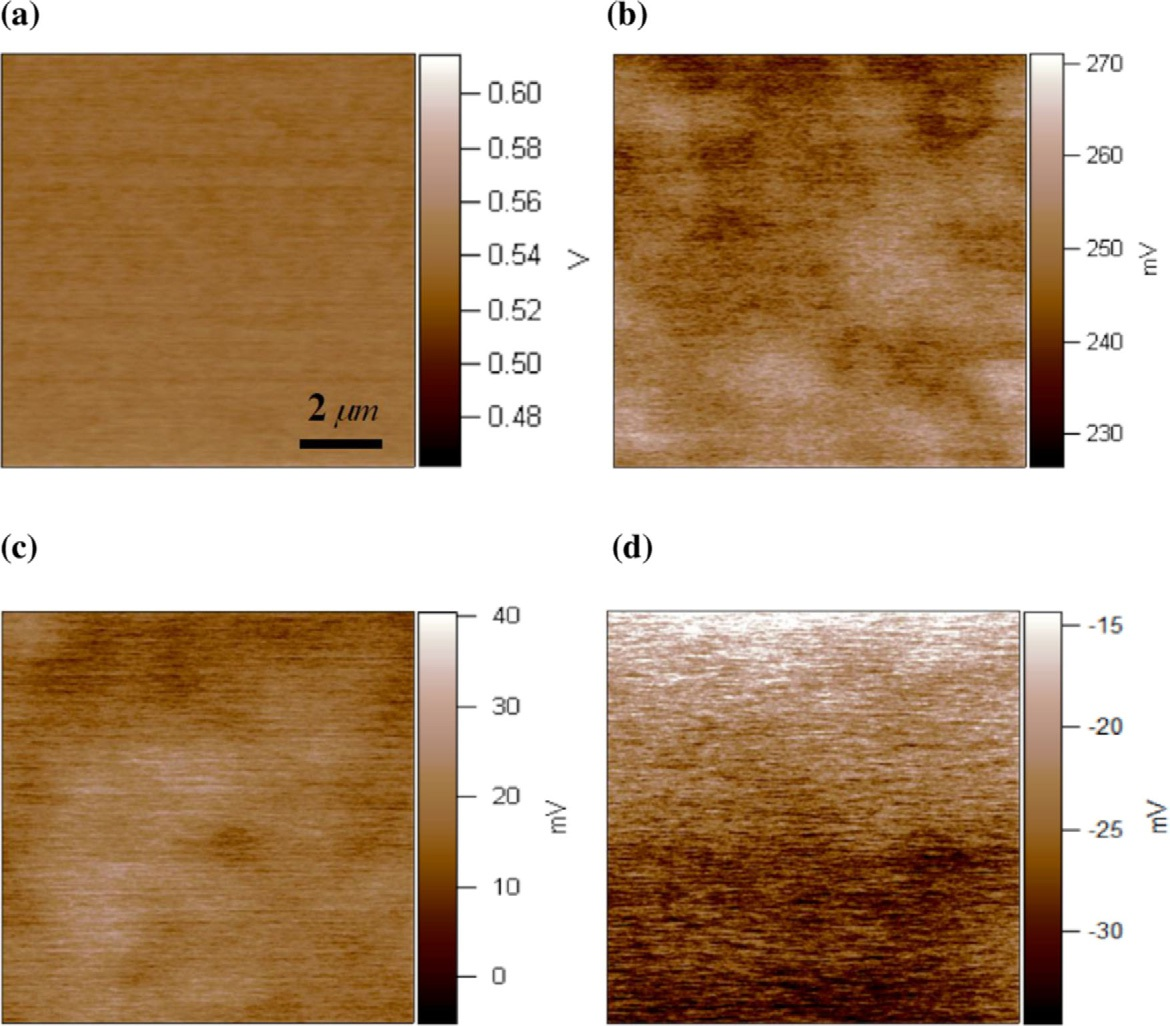
KPFM images of a) the undoped ZnO film, b) 2 % Cu‐doped ZnO film, c) 8 % Cu‐doped ZnO film, and d) 10 % Cu‐doped ZnO film. The surface potentials were 550 and 18 mV for the ZnO film and 8 at % copper‐doped ZnO film, respectively. (Figure taken from Ref. 81, with permission.).

Finally, nonvolatile random access memory devices are able to retain information if the power is turned off. Ferroelectric thin films are suitable candidates to build such memory devices. Su and Zhang used KPFM to investigate the surface potential dependence on voltage and duration upon application to copper‐doped ZnO films (see Figure 6).^81^ They concluded that the copper‐doped ZnO films exhibited enhanced bipolar charge‐trapping properties, which could be an advantage for nonvolatile memory applications. The difference can be understood in terms of the doping‐induced shift of the Fermi level, which provides a good representation of the characteristics of the semiconductor material.

## 4 Summary and Outlook

In the last three decades, atomic force microscopy (AFM) has expanded its use to many scientific and technological fields. This short Review has presented four measuring modes based on AFM. Although the described methods have been successfully applied to study different problems, there is room for improvement, for example, high‐speed imaging has opened up new possibilities for exploration (as pointed out before, this alone should be the topic of a separate review). The appropriate model to interpret mechanical experiments performed on soft‐matter systems is still required. In addition, a critical issue for tip‐enhanced Raman spectroscopy (TERS) experiments is optimal tip preparation that could ensure good reproducibility and high enhancement. Another issue is spectral interpretation and the difficulty in locating characteristic bands that are specific to each sample.

Kelvin‐probe force microscopy (KPFM) has not yet been widely utilized in biological and colloidal systems, mainly because it is used in air. A difficult challenge would be to elucidate the surface potential in biosystems because it is affected by interactions with the medium. This task is certainly relevant because surface potentials are important for the interaction of particles, biointerfaces, cells and tissues.

Finally, a summary of the main properties and challenges associated with AFM, KFPM, and TERS is provided in Table 1.

**Table 1:** Summary of the measuring properties/advantages and drawbacks/challenges of AFM, KFPM, and TERS.

Device	Measuring properties/advantages	Drawback/challenges
AFM^[a]^	functions in air, vacuum, and liquid (between ≈5 and 60 °C); atomic resolution in high vacuum (sub‐nanometer in liquid); in general, lateral molecular resolution (≈1–5 nm) and sub‐nanometer vertical resolution (≈1 nm) 3D surface profile to deliver direct information about the interaction forces between tip and sample; useful for studying mechanical properties of biomaterials at the nano‐ and microscale; does not require either vacuum or possibly damaging sample treatment (e.g., metal/carbon coatings)	smaller scan image size (μm range) compared with scanning electron microscopy (mm range); slow scan time could lead to thermal drift (partially solved with high‐speed AFM); AFM images can be affected by piezoelectric hysteresis (closed‐loop scanners might eliminate this problem); unsuitable tips can produce image artifacts
KFPM^[b]^	simultaneous mapping of topography and potential (or work function); mainly used for materials science for metallic and semiconducting structures; allows measurement of the contact potential difference with lateral and potential resolutions below 50 nm and 10 mV, respectively;	surface potential control is important for materials and biological systems (e.g., crucial for the interaction of living cells with exogenous devices); surface potential while studying biological samples is affected by the environmental medium (and surface contaminants); measurement in liquid requires AFM tips insulated at their apex to prevent current leakage; proper comparison and interpretation of KFPM data with results obtained from electrophoretic mobility and streaming potential; cheap and robust electrode fabrication
TERS^[c]^	nanometer spatial resolution (≈30 nm); chemical fingerprint recognition (identification and classification of materials); there are still unresolved questions about mode selectivity (this might limit certain applications); could benefit from the ability to use visible light for excitation	ability to investigate samples in solutions; tip reproducibility and reference samples; deeper understanding of the TERS process; statistical data analysis

[a] Introduced by Binnig and co‐workers in 1986.^1^ [b] Introduced by Nonnenmacher and co‐workers in 1991.^82^ [c] Introduced by Stöckle and co‐workers^83^ and Anderson^84^ in 2000.

## Conflict of interest

*The author declares no conflict of interest*.

## Biographical Information

José L. Toca‐Herrera received his degree in physics from the University of Valencia. He spent a year on research training at the Max‐Planck Institute for Polymer Research and completed his Ph.D. at the Max‐Planck Institute of Colloids and Interfaces under the supervision of Prof. Helmuth Möhwald. After postdoctoral stays at the Technical University of Berlin, University of Cambridge, and BOKU‐Vienna, he started at Rovira i Virgili University as RyC Research Professor and later moved to CIC‐BiomaGUNE. In 2010, he joined BOKU‐Vienna as Full Professor where he leads the Institute for Biophysics. His research includes soft matter, colloids, interfaces, and mathematical methods. He has been Visiting Professor at the Max‐Planck Institute of Colloids and Interfaces and AGH‐University.